# Touting for Quacks

**Published:** 1913-01

**Authors:** 


### BUFFALO MEDICAL JOURNAL

**A Monthly Review of Medicine and Surgery**

*EDITOR*

A. L. BENEDICT.

All communications, whether of a literary or business nature, books for review and exchanges, should be addressed to the editor, **228 Summer Street, Cor. Elmwood Avenue, Buffalo, N. Y. Subscribers, advertisers and exchanges please revise address.** Please make personal or telephonic calls before 1 p. m.

THE BUFFALO MEDICAL JOURNAL will publish regularly. full transactions of any professional organization in western New York at cost. Personal notices, brief reports of society proceedings, and the like, are always welcome and will be published without charge.

Subscriptions may commence at any time. $2.00 per year in advance.

VOL. LXVIII. JANUARY, 1913 No. 6

### Touting for Quacks

A physician who had recently lost a member of his family was pained to receive, shortly after his death, a circular letter addressed to the patient, and offering treatment by mail. This circular came from a distant city. The regularity with which advertising matter of this sort is addressed to the sick, indicates a systematic canvass for victims. It is scarcely imaginable that lists of names can be secured from attending physicians—certainly not in a case of the kind mentioned—nor that reports to boards of health are thus exploited—and, in the present instance, the case was not reportable. We strongly suspect that some of the advertisements of nice, light, remunerative work for ladies (not canvassing), refer to ferreting out of cases for quacks. Perhaps an investigation of the methods of obtaining address lists for quacks and popular education on the subject might be worth while.

